# Onion-like multilayered polymer capsules synthesized by a bioinspired inside-out technique

**DOI:** 10.1038/s41467-017-00077-7

**Published:** 2017-08-04

**Authors:** Brady C. Zarket, Srinivasa R. Raghavan

**Affiliations:** Department of Chemical & Biomolecular Engineering, University of Maryland, College Park, Maryland 20742 USA

## Abstract

Diverse structures in nature, such as the spinal disc and the onion have many concentric layers, and are created starting from the core and proceeding outwards. Here, we demonstrate an inside-out technique for creating multilayered polymer capsules. First, an initiator-loaded gel core is placed in a solution of monomer 1. The initiator diffuses outward and induces polymerization, leading to a shell of polymer 1. Thereafter, the core-shell structure is loaded with fresh initiator and placed in monomer 2, which causes a concentric shell of polymer 2 to form around the first shell. This process can be repeated to form multiple layers, each of a distinct polymer, and of controlled layer thickness. We show that these multilayered capsules can exhibit remarkable mechanical resilience as well as stimuli-responsive properties. The release of solutes from these capsules can be tailored to follow specific profiles depending on the chemistry and order of adjacent layers.

## Abstract (web summary)

Multiple concentric layers are present in a variety of structures present in nature, including the onion. Here, the authors show an inside-out strategy to synthesize multilayered polymer capsules, with different layers having specific composition and thereby specific responses to stimuli such as pH and temperature.

## Introduction

Nature is increasingly providing the inspiration for the design of new materials^1–7^. Significant efforts have been devoted to mimicking the microstructure or nanostructure found in natural materials like opals, nacre, gecko feet, bird beaks, etc^4–7^. However, the large-scale (i.e., over mm to cm) structure of natural materials can also be very interesting and provide a source of inspiration. Consider the examples of a plant seed, an egg, a spinal disc, and an onion^8–10^. A common theme to all these materials is that they have many different layers, roughly arranged in a concentric fashion. In the case of an egg (and embryos in general), the yolk and the genetic material form the inner core, and this is surrounded by the albumen, then multiple protein membranes, and finally the inorganic outer shell^8^. Many tissues and body parts are also multilayered^8, 9^. For instance, the spinal discs located between consecutive vertebrae in the spine have two layers: a soft core surrounded by a stiffer shell^9^. One final example is that of an onion, which has a developing bud in the center, followed by many water-rich concentric layers, and a drier outer scale^10^. A key point from these examples is that the concentric layers in a given material often have different composition, which in turn dicates their distinct function in the overall material. Can this design principle be adapted for the design of new materials? That is, can we create materials having multiple layers, each of a different composition and thereby different properties?

In addition to structure, another noteworthy aspect of natural materials deals with the manner of their growth. The growth of a specific structure and shape in nature is termed morphogenesis^11, 12^. To form a multilayered structure, such as an egg, it is evident that the core must form first, followed by the next several layers, and finally the outer shell. Moreover, natural growth invariably occurs from the inside-out^11^. That is, not only does the core form first, but it can dictate the subsequent growth, which occurs in a direction radiating outward from the center. For instance, consider how a seed (or an egg) develops into a full-fledged organism^10^. The growth begins at the surface of the seed and proceeds radially outward, utilizing nutrients from the external medium. Importantly, the seed (core) controls the growth rate and extent. This strategy is fundamentally different from common processes used in materials synthesis, such as nucleation-and-growth, self-assembly, or additive manufacturing^7, 13^. In nucleation-and-growth, for example, nuclei can grow outward to form macroscopic crystals, but the rate and form of growth is controlled by the availability of external precursor, not by the core nucleus^13, 14^. In additive manufacturing (3-D printing), macroscopic objects can be formed by adding one layer of material at a time, but this is basically a deposition scheme controlled from the outside; thus the core of the object does not dictate the growth^15^. To our knowledge, true inside-out strategies have rarely been exploited in materials design, especially in the context of soft materials.

Here, we describe the synthesis of multilayered polymer capsules, for which we have developed an inside-out strategy. Polymer capsules are structures in which a polymeric shell surrounds a liquid core^16, 17^. Such capsules are used to store and release solutes, and they find a variety of applications ranging from cosmetics to drug-delivery^16, 18, 19^. Research in this area has focused on stimuli-responsive capsules, where the release of solutes can be modulated by an external trigger, such as temperature, pH, or light^18, 19^. Recently, capsules have also been synthesized with many concentric, but identical, layers^20–28^. However, to our knowledge, there have been no reports in which diverse polymeric layers (shells) are integrated together in a capsule. This was our focus, and we demonstrate that a strategy involving successive free-radical polymerizations around an initial gel core can lead to multiple layers with very different composition and properties. The key in our strategy is that the initiator for the polymerization is present only in the core, and therefore layer growth is controlled by the diffusion of initiator from this core (hence the term ‘inside-out’ for this strategy). Significantly, both the thickness and composition of each layer can be independently tuned. We particularly highlight the interesting cases where one (or some) of the polymeric layers are responsive to a stimulus, viz. pH or temperature. Solute release from such responsive capsules is shown to follow step-like (pulsatile) profiles, which suggests their potential utility in delivery applications^29–31^. More generally, our strategy can be used to create new multifunctional materials that mimic the remarkable structures found in nature.

## Results

### Synthesis of multilayer capsules

**Fig. 1:**
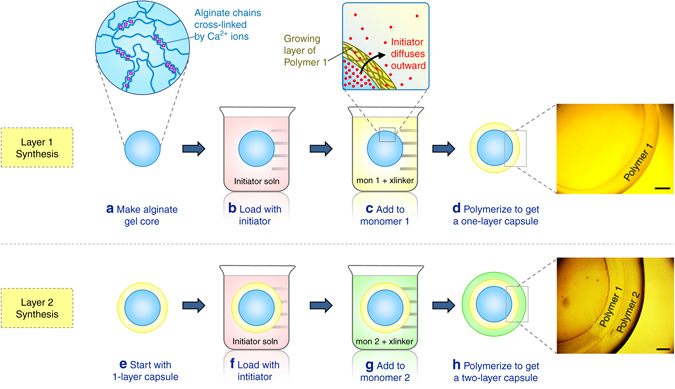
Synthesis of multilayer capsules. An alginate gel core is first made (**a**) and this is loaded with free-radical initiator (**b**). The gel is then introduced into a solution of monomer 1 (along with cross-linker and accelerant) (**c**). Upon polymerization, a layer of polymer 1 is formed around the gel core (**d**). The *inset* in **c** shows that this layer is formed by diffusion of intitator outward from the core into the monomer solution. This process is then repeated with the one-layer capsule (**e**), which is loaded with initiator (**f**) and then contacted with monomer 2 (**g**). Upon polymerization, a second layer of polymer 2 is formed (**h**). The process can be further repeated to introduce additional layers. Scale bars are 500 µm

We present a step-wise technique for generating polymeric multilayer capsules, as shown schematically in Fig. 1. First, a gelled core is created by the physical cross-linking of a biopolymer. We have used many biopolymer gels for this purpose, including those based on chitosan, gelatin, and agarose^32^. But for most of the studies described in this paper, the cores are made from the biopolymer, alginate^33, 34^. To create the gelled cores, a solution of 2 wt% sodium alginate is added drop-wise to a solution of 0.5 M calcium chloride (CaCl_2_) using a syringe (Fig. 1a). The alginate droplets become cross-linked by the Ca^2+^ ions into gelled beads, with the bead diameter typically being 0.5–5 mm. The *inset* in Fig. 1a shows the structure of this alginate gel; note that the Ca^2+^ ions form junctions between alginate chains^34^. The alginate bead is then loaded with ammonium persulfate (APS), which is a water-soluble initiator for free-radical polymerization. For this, the bead is incubated in a solution of 15 mg/ml initiator for at least 10 min (Fig. 1b). The incubation time was set at 10 min based on a calculation from Fick’s 2nd law for diffusion, which revealed that this time was ample for the center of a 4-mm bead to equilibrate to roughly the bulk concentration^35^.

Next, the initiator-loaded gel is transferred to a solution containing a monomer (labeled monomer 1 in Fig. 1c) such as N-isopropyl acrylamide (NIPA) at a 1 M concentration, a cross-linker like N,N′-methylenebis(acrylamide) (BIS) at 2.2 mol% with respect to the monomer, and accelerant. Free-radical polymerization is then conducted at room temperature (Fig. 1d). Polymerization begins as the persulfate ions diffuse from the core into the surrounding solution and react with the monomers. A layer (shell) of cross-linked polymer thus forms around the core. As shown in the *inset* to Fig. 1c, the polymer layer grows in a radial direction outward from the core because of the diffusion of initiator from the core. That is, the initiator concentration is highest at the surface of the core and decreases in a radial direction towards the bulk solution^35^. We term this growth an inside-out process. Once a layer of sufficient thickness has formed (typically this takes ~10 min), we can remove the structure, wash it with water and store it in water or buffer. At this point, we have a gelled core surrounded by a layer of polymer 1 (NIPA), which can be clearly seen in the *inset* image.

**Fig. 2:**
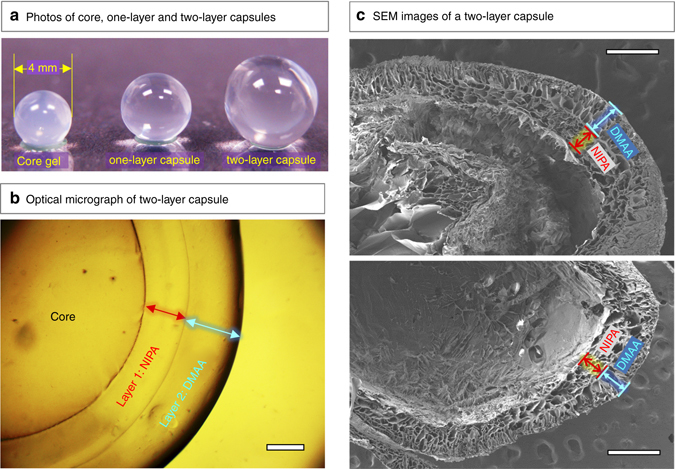
Images of multilayer capsules at different length scales. (**a**) Photos of the alginate gel core and the corresponding one-layer and two-layer capsules. (**b**) Optical micrograph of a capsule with an alginate (Alg) core, an inner layer of N-isopropylacrylamide (NIPA) and an outer layer of N,N′-dimethylacrylamide (DMAA). The overall structure is denoted as Alg–NIPA–DMAA. The scale bar is 500 µm. (**c**) Scanning electron micrographs of two Alg–NIPA–DMAA capsules (after freeze-drying). The boundaries between the layers can be distinctly seen in both cases. Scale bars in the images are 500 µm.

The above process is repeated to form a layer of a second polymer, as shown in Fig. 1. For this, we reload the above structure with APS initiator and place it in a solution of monomer 2, i.e., N,N′-dimethylacrylamide (DMAA), along with the same BIS crosslinker and accelerant. A second polymerization step then yields a second concentric layer of polymer 2 (Fig. 1h). The second layer grows from the surface of the first layer, again consistent with inside-out growth. We now have a spherical capsule with an alginate core (Alg), then a surrounding layer of polymer 1 (NIPA), and finally an outer layer of polymer 2 (DMAA), as shown in the image in Fig. 1h. This capsule is designated as Alg–NIPA–DMAA, which signifies the order of layers outward from the core. The same process can be further repeated to give additional concentric layers of different polymers. Also, the alginate core in the capsule can be ungelled to form a liquid core. This can be done by immersing the capsule in a solution of a calcium chelator like sodium citrate or ethylene diamine tetracetic acid (EDTA)^33^. For most of the experiments described below, however, we have left the core intact. For other gelled-cores made from gelatin or agarose, the biopolymer gels are thermoresponsive and hence the core gels can be liquefied by moderate heat^32^.

Fig. 2a shows the alginate core next to a single-layer capsule and a two-layer capsule generated by sequential polymerization. The alginate core has a diameter of 4 mm. The first surrounding layer is a network of NIPA (~550 µm thick) and the second layer is a network of DMAA (~750 µm thick). The optical microscope image (Fig. 2b) clearly shows the presence of two distinct layers that are not interpenetrated. Scanning electron microscopy (SEM) images of such Alg–NIPA–DMAA capsules after freeze-drying (Fig. 2c) further confirm the discrete nature of the two layers. Each layer appears porous in these images, which is consistent with their being polymer networks. The pores appear to be oriented along slightly different directions, allowing the layers to be distinguished. Note that the SEM images are of two capsules with identical layer composition but synthesized separately. The similar microstructure in both cases shows that these multilayer capsules can be reproducibly synthesized. Overall, the presence of multiple concentric layers in our capsules is reminiscent of natural multilayered materials like the onion. Supplementary Fig. 1 illustrates the structure of different layers present in a plant seed, an egg, a spinal disc, and an onion.

**Fig. 3:**
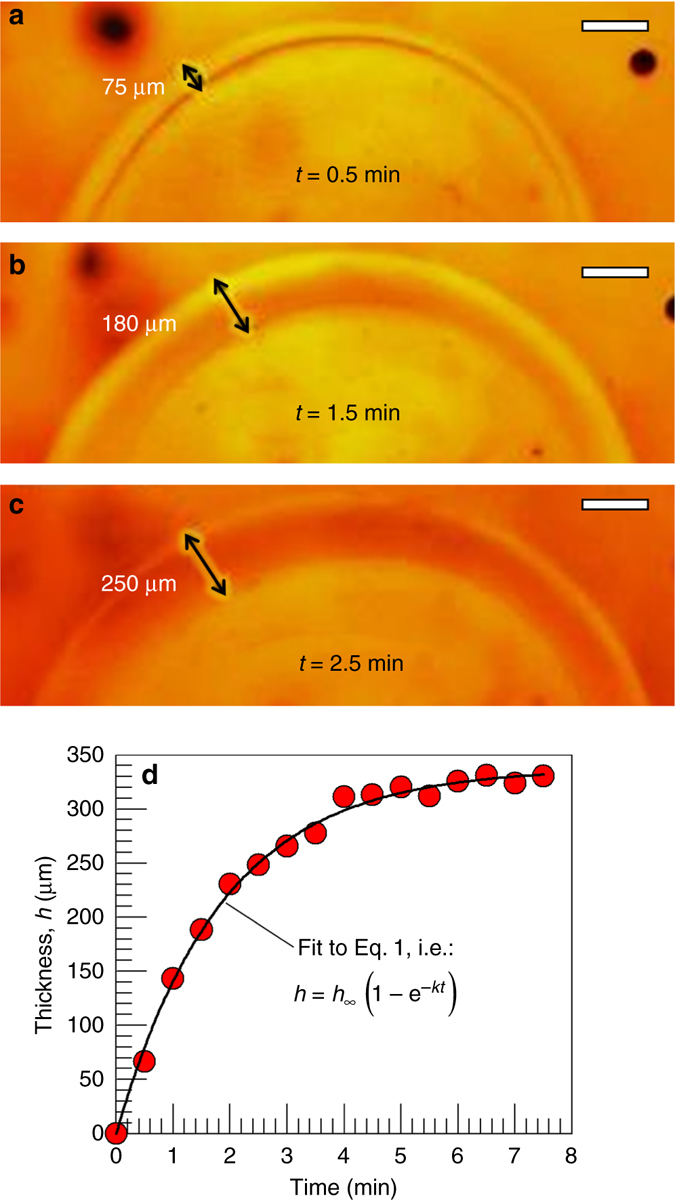
Kinetics of layer growth, visualized directly by optical microscopy. At time *t* = 0, an alginate gel core of 2 mm diameter, loaded with 15 mg/ml ammonium persulfate (APS) initiator, is placed in a solution of 1 M DMAA monomer (together with cross-linker and accelerant). Still images at various time points are shown in **a**, **b**, and **c**, and these reveal the growth of the polymer layer around the core. Scale bars in the images are 200 µm. In **d**, a plot of the layer thickness *h* vs. *t* is shown, and the solid curve through the data is a fit to Eq. 1

The above procedure can be used to synthesize multilayer capsules over a range of sizes. We have varied the diameter of biopolymer cores over approximately two orders of magnitude: from ~10 mm to about 200 µm. Optical images of single-layer capsules over this size range are shown in Supplementary Fig. 2. Note that, to create biopolymer gel cores with diameters <1 mm, we resorted to a microfluidic technique developed in our laboratory in which pulses of compressed gas are used to shear off biopolymer-bearing aqueous droplets from the tip of a capillary^36, 37^. Once cores of a given size are created, the rest of the procedure is the same as shown in Fig. 1, i.e., the cores are loaded with APS initiator, then placed in a solution of monomer, cross-linker and accelerant. The images in Supplementary Fig. 2 show that a polymer shell is formed around the core in all cases.

### Kinetics of layer growth

As mentioned, each polymer layer in the capsule grows from the inside out, and we now show that this growth can be visualized in real time. For this, we prepared an alginate core of 2-mm diameter and loaded it with 15 mg/ml of APS initiator. At time *t* = 0, we placed this sphere in a solution of 1 M DMAA with cross-linker and accelerant. The sphere was then observed by optical microscopy (Fig. 3). Based on the images, a layer of polymer can be discerned around the core within 30 s (Fig. 3a), and as time progresses, this layer grows outward. The layer thickness *h* at each time point can be extracted from the images, and this quantity is plotted vs. time *t* in Fig. 3d. Note that within about 8 min, the growth of the layer is complete and it saturates at a value around 338 µm. Even after a period of 24 h, the layer thickness remains at this steady-state value *h*_∞_. We can then fit the *h*(*t*) data to the following functional form:^35^

1 $$h = {h_\infty }\left( {1 - {{\rm e}^{ - kt}}} \right)$$

The only adjustable parameter in the above equation is the effective rate constant *k*. Equation 1 gives a very good fit to the data in Fig. 3d with *k* = 0.54. Note that *k* accounts for the combination of two steps occurring in series: mass transfer (i.e., diffusion) of initiator from the core into the external solution, followed by the kinetics of the polymerization reaction^35^.

Based on the above results, the layer thickness *h* around a given core can be fixed by arresting the polymerization at a particular time, i.e., by replacing the monomer-laden solution with water at this time. Alternately, the layer thickness at steady-state *h*_∞_ can be varied systematically by modulating the reaction kinetics. The parameters that affect the kinetics include: the concentration of initiator in the core; the concentrations of monomer and cross-linker in the external solution; the reaction temperature; and the viscosity of the external solution^38^. A detailed study on these parameters is beyond the scope of this initial paper. But as an example, we have varied the concentration of APS initiator. Using an identical setup to the one above, we reduced the APS in the core from 15 mg/ml to 7.5 and 3.75 mg/ml. All other conditions were kept the same and the polymerization was conducted for 24 h in each case to allow the layer-thickness to reach steady-state. We found that reducing the initiator decreases the layer thickness: while *h*_∞_ was 338 µm for 15 mg/ml of APS in Fig. 3d, reducing the APS by a factor of 4 to 3.75 mg/ml resulted in a drop in *h*_∞_ to ~90 µm.

### Mechanical properties

**Fig. 4:**
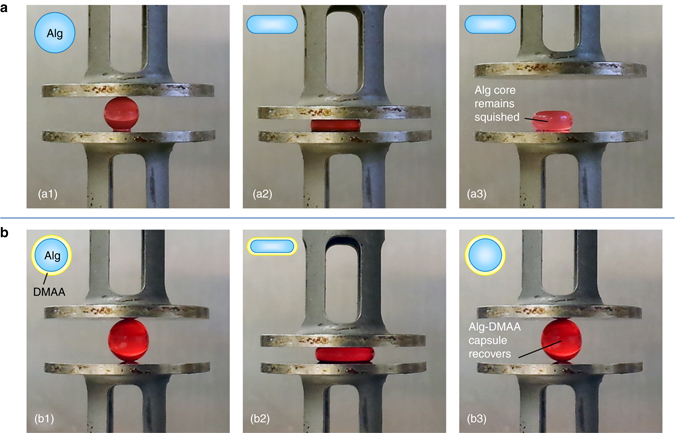
Contrasting mechanical properties of a core gel particle versus a single-layer capsule. Photos are shown of an Alg gel particle (**a**) and an Alg–DMAA capsule (**b**) being compressed between parallel plates. Both have the same core diameter of 4.6 mm, with the DMAA shell being 200 µm thick. Photos a1 to a3: when the Alg gel is compressed by 50%, it remains squished and does not recover when the compression is removed (plastic response). This is indicated by the schematics and also demonstrated by Supplementary Movie 1. Photos b1 to b3: when the Alg–DMAA capsule is compressed by 60%, it recovers as soon as the compression is removed (elastic response). Thus, the addition of the thin DMAA shell (1/20th the thickness of the core) dramatically alters the mechanical properties. This behavior is indicated by the schematics and also shown by Supplementary Movie 2, which further shows that the elastic behavior is preserved over multiple cycles of compression

Our multilayered capsules tend to have very different mechanical properties compared to their gel cores. The properties depend on the composition of each layer and on the number of layers. While a detailed study on these aspects is beyond the scope of this paper, we highlight a couple of aspects here. Most importantly, the addition of even a thin shell to a core can radically alter its mechanical response. This is best demonstrated in tests under compression, as shown in Fig. 4. Here, we contrast an Alg core and an Alg–DMAA capsule with compositions identical to those described above. The Alg core is a gel of diameter 4.6 mm. The Alg–DMAA capsule is created with an identical Alg core and adding a DMAA shell of thickness 200 µm (i.e., 0.2 mm) to it; the shell is thus 1/20th the diameter of the core, i.e., it is very thin in comparison. The behavior in Fig. 4 is also depicted in two movies (Supplementary Movies 1 and 2), which are provided in the Supplementary Information section.

First, consider the Alg gel core (Photos a1 to a3 in Fig. 4). When this is compressed, the initial sphere is squished into an ellipsoidal (disc or pancake) shape. If the compressive strain is 50% or more, then the sphere remains squished as a disc (Photo a3). In other words, the gel suffers a plastic (irreversible) deformation when compressed, and this can be further confirmed by Supplementary Movie 1. Indeed, such a response is familiar to researchers who work with gels of biopolymers like alginate, and it is known that these gels have limited mechanical resilience^34, 39^. Next, consider the response of the Alg–DMAA capsule (Photos b1 to b3 in Fig. 4). In this case, even when the initial sphere is compressed by 60%, it still recovers to its initial size after the compression. That is, the deformation is reversible, and the response is elastic. Supplementary Movie 2 shows the capsule being subjected to three successive compression-recovery cycles, and after each cycle, the capsule recovers its original size. The above behavior can be easily confirmed by taking these objects and squeezing them between one’s fingers. We consistently find that the Alg cores are squishy and plastic while Alg–DMAA capsules respond as elastic objects. In short, adding a thin shell to the gel core makes it much more resilient and elastic.

We have attempted to quantify the differences seen in Fig. 4. For this, we placed the above structures between the plates of a rheometer and compressed them at 10% strain/min^40, 41^. The compressive stress was measured and is plotted in Supplementary Fig. 3a for the Alg core and in Supplementary Fig. 3b for the Alg–DMAA capsule. These data confirm the visual observations. That is, during the first loading cycle, the Alg core gets irreversibly compressed. When the stress is released, the top plate detaches from the sample, and the stress therefore plummets to zero. The Alg–DMAA capsule, on the other hand, can be subjected to multiple compression-recovery cycles, which reflects its elastic response (akin to a cross-linked rubber). Note that the non-linearity of the response makes it difficult to extract an elastic modulus from the initial portion of the data. Further analysis, including comparisons of the modulus, the strain at break, and the compressive strength will be reported in a future paper. We briefly mention two other points of interest here. First, the failure mode of the capsule is also distinct from that of the core. That is, when compressed beyond a critical strain, the Alg core ruptures into many pieces^42^, whereas the Alg–DMAA capsule suffers a break in its shell, with the core then ejecting out as a distinct entity. Secondly, the elastic nature of the capsule is also reflected in its ability to bounce off a hard surface. That is, the Alg–DMAA capsule bounces to a greater height compared to the Alg core, i.e., its coefficient of restitution is much higher.

### Stimuli-responsive layers

**Fig. 5:**
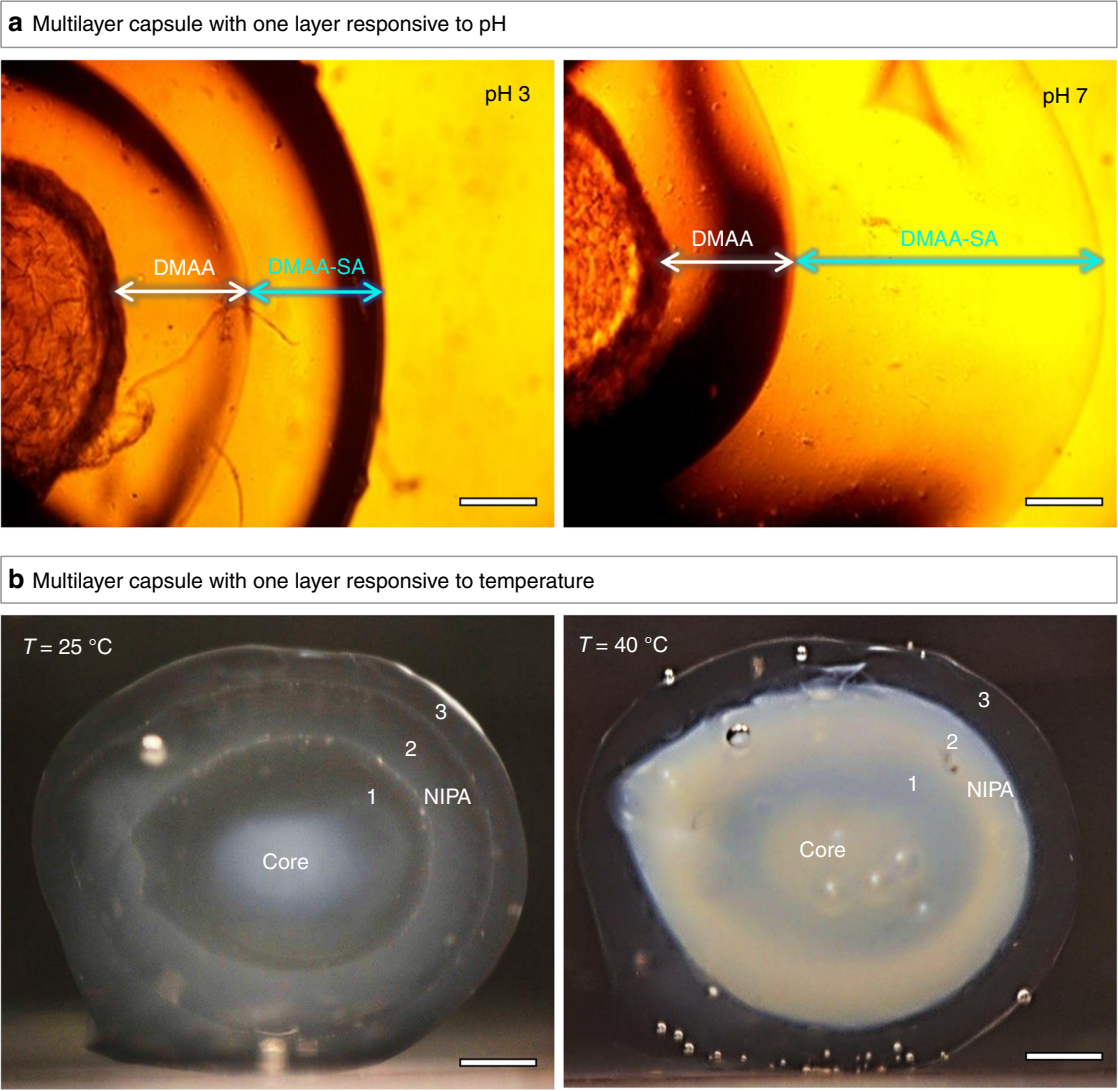
Multilayer capsules with specific layers responsive to external stimuli. In **a**, a two-layer capsule is shown with an inner layer of nonionic polymer (DMAA) and an outer layer of anionic polymer, obtained by copolymerization of DMAA with sodium acrylate (SA) (designated as DMAA–SA). At pH 3, the two layers have the same thickness. At pH 7, the carboxylate groups in the DMAA–SA layer become deprotonated, causing the anionic gel to swell, and thus the thickness of the DMAA–SA layer increases substantially. In **b**, a three-layer capsule is shown, where layers 1 and 3 are DMAA (non-responsive), while layer 2 is NIPA (thermoresponsive). At ambient temperature (25 °C), all layers are transparent gels. Upon heating to 40 °C, which is above the lower critical solution temperature (LCST) of NIPA, the NIPA layer becomes turbid. Scale bars are 500 µm in **a** and 1 mm in **b**

A key feature of our synthesis scheme is that it allows integration of different polymeric layers into the same capsule. The interesting combinations are when one (or more) of the layers are responsive to external stimuli while others are not. We present two examples to illustrate these capabilities. First, we consider pH as a stimulus. It is well-known that ionic polymer gels exhibit a different response to pH compared to nonionc polymer gels^43–45^. For example, an anionic gel based on a monomer such as sodium acrylate (SA) will be swollen at high pH when all its carboxylate groups are ionized and shrunken at low pH when the same groups lose their charge^46–48^. Nonionic gels, on the other hand, will exhibit the same volume at low and high pH. These differences are highlighted by a two-layer capsule in Fig. 5a where the inner layer is nonionic while the outer layer is anionic. To make this capsule, we first created a pH-insensitive core of chitosan (an amine-rich polysaccharide) cross-linked with glutaraldehyde (GA)^36, 49^. The core was made as before by adding the chitosan solution drop-wise into a solution of the GA; note that GA forms covalent bonds between the amines on chitosan^32^. We then polymerized a layer of nonionic DMAA around this core by the procedure described earlier (Fig. 1). Next, we polymerized an anionic layer around the first layer. For this, the capsule from the previous step was loaded with initiator and placed in a solution containing DMAA and SA (at a molar ratio 9:1) as well as crosslinker (BIS) and accelerant. Figure 5a shows an image of the the two-layer capsule in a pH 3 solution. In this case, the two layers have about the same thickness, i.e., ~900 µm. This is because the carboxylate groups of SA are not ionized under acidic conditions. Next, Fig. 5a shows the same capsule in a pH 7 solution. While the inner DMAA layer remains at the same thickness as at pH 3, the outer DMAA–SA layer is now swollen to about 2000 µm, which is an increase by more than 100%. This illustrates the pH-responsive properties of our multilayer capsule.

Next, we consider temperature as a stimulus. NIPA is well-known to be a thermoresponsive polymer, i.e., NIPA gels shrink when heated above the polymer’s lower critical solution temperature (LCST) of 32 °C^45, 50^. DMAA, on the other hand, is not affected by temperature^48^. Figure 5b shows a three-layer capsule where layer 1 and 3 are DMAA and layer 2 is NIPA (the core is alginate/Ca^2+^, as before). The photo taken at 25 °C clearly shows all three layers, with each layer being about 1 mm thick. Next, a photo is shown of the same capsule after heating to 40 °C, which is above the LCST of NIPA. In this case, the NIPA layer (2) has become opaque, and this is because the NIPA chains turn hydrophobic above the LCST and the gel begins to expel water^35, 40^. In contrast, the DMAA layers (1 and 3) are unaffected by temperature and are actually both clear (the inner layer 1 of DMAA may seem somewhat turbid, but that is only because it is obscured by the surrounding NIPA layer 2). Overall, the above capsule shows a visible macroscopic change in response to temperature.

### Solute release from temperature-responsive capsules

We now study the release of small-molecule solutes from the above kind of temperature-responsive capsules. As mentioned earlier, capsules are frequently used for the delivery of drugs and other solutes^18, 19^. In this context, the proximity of NIPA’s LCST to human body temperature (37 °C) has made this polymer of particular interest in drug delivery^45^. For example, a number of groups have demonstrated pulsatile release of drugs from thermosensitive NIPA gels through temperature control^29–31^. Inspired by these past studies, we investigated whether the multilayer structure of our capsules could make them interesting candidates for drug delivery. For these experiments, we worked with two-layer capsules having concentric layers of DMAA and NIPA, but in different order.

**Fig. 6:**
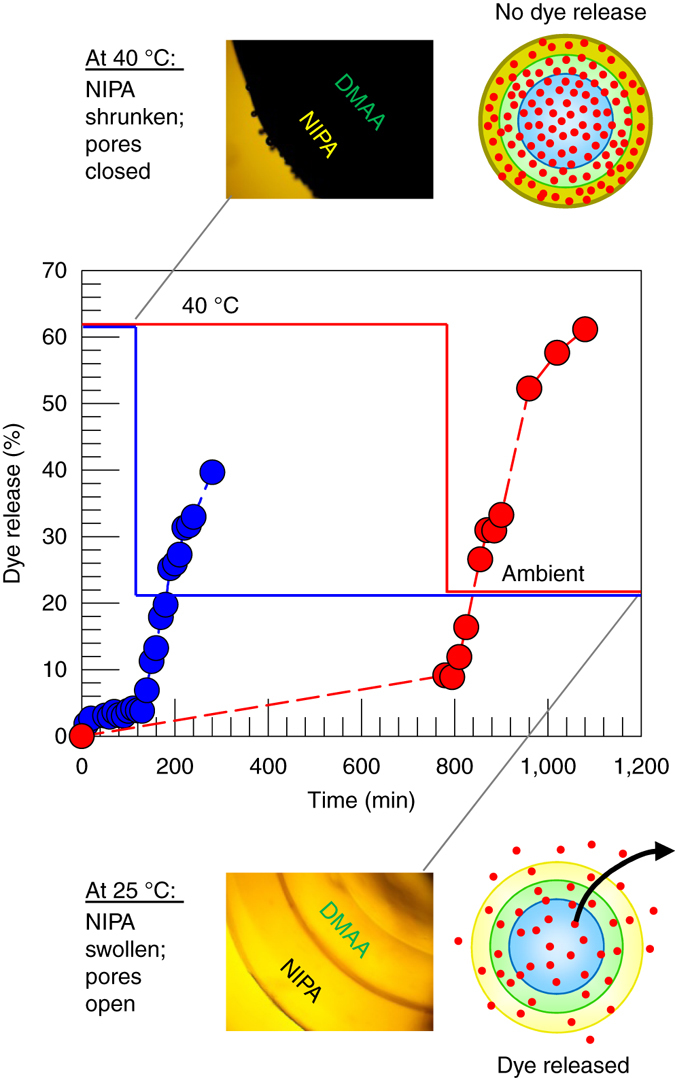
Temperature-responsive release of dye from a two-layer DMAA–NIPA capsule. Here, DMAA is the inner layer and NIPA is the outer layer. At 40 °C (above the LCST of NIPA), the pores in the outer NIPA layer are closed; thus, the dye remains in the capsule. This is indicated by the schematic, and the corresponding micrograph shows a dark capsule due to the turbidity of the outer layer. After a certain time (110 min for the *blue curve*; 780 min for the *red curve*), the temperature is lowered to ambient (25 °C). The pores in the NIPA layer open up, causing the dye to release. This is shown by the schematic, and in this case the micrograph shows a transparent capsule. The *y*-axis is normalized to the dye released into solution after a long time (2 days)

First, we consider an Alg–DMAA–NIPA capsule, i.e., with DMAA as the inner layer and NIPA as the outer layer. This capsule was loaded with brilliant yellow (BY) dye by soaking in a 500-µM dye solution for 24 h at room temperature. The capsule was then heated in the dye solution up to 40 °C, a temperature that exceeds the LCST of NIPA. This causes the outer NIPA layer to shrink, thereby preventing release of dye from the inner portions of the capsule (see more details below). Next, the capsule was rinsed briefly with deionized (DI) water at 40 °C, and then transferred to a flask maintained at 40 °C and containing 100 ml of DI water. The dye concentration in the external solution was then monitored as a function of time, and this is plotted in Fig. 6. As long as the temperature is at 40 °C, we find negligible dye to be released from the capsule, with the % release (*blue curve*) saturating at ~5%. Next, at the 110 min mark, we stopped heating the flask and allowed it to cool to ambient temperature. As the temperature drops below the LCST of NIPA, we observe a sharp increase in dye release. Within the next 180 min (3 h), more than 40% of the dye gets released. Note that the *y*-axis in Fig. 6 is normalized to the dye in the solution 2 days later at ambient temperature, i.e., at a state when all the dye has been released from the capsule.

A similar experiment was repeated, where an identical capsule to the above was held at 40 °C in the flask for 13 h (780 min). Even over this longer period (*red curve* in Fig. 6), only about 10% of the dye gets released. When heating is stopped and the system is cooled, the dye release is again triggered and over the next 300 min, more than 60% of the dye is released. A key result from these experiments is that small-molecule solutes can be kept encapsulated for extended periods of time by exploiting the thermoresponsive properties of NIPA. When a NIPA gel is heated above its LCST, its chains become hydrophobic and the gel becomes turbid^35, 38^. Similarly, when the Alg–DMAA–NIPA capsule is observed under a microscope at 40 °C, the capsule appears dark (see image in Fig. 6) because the NIPA layer is turbid and it is the outer layer. We believe the hydrophobic NIPA chains close the pores in the NIPA layer, akin to forming a precipitate around the pores. This allows the dye to be retained in the core. When temperature is decreased to 25 °C, the capsule becomes clear again (see image in Fig. 6). In this state, the pores in the outer NIPA layer are reopened, allowing the dye to diffuse out. Overall, Fig. 6 shows that we can engineer a one-step release profile, i.e., with no release under one value of the stimulus, followed by rapid release under a different value of the same stimulus.

**Fig. 7:**
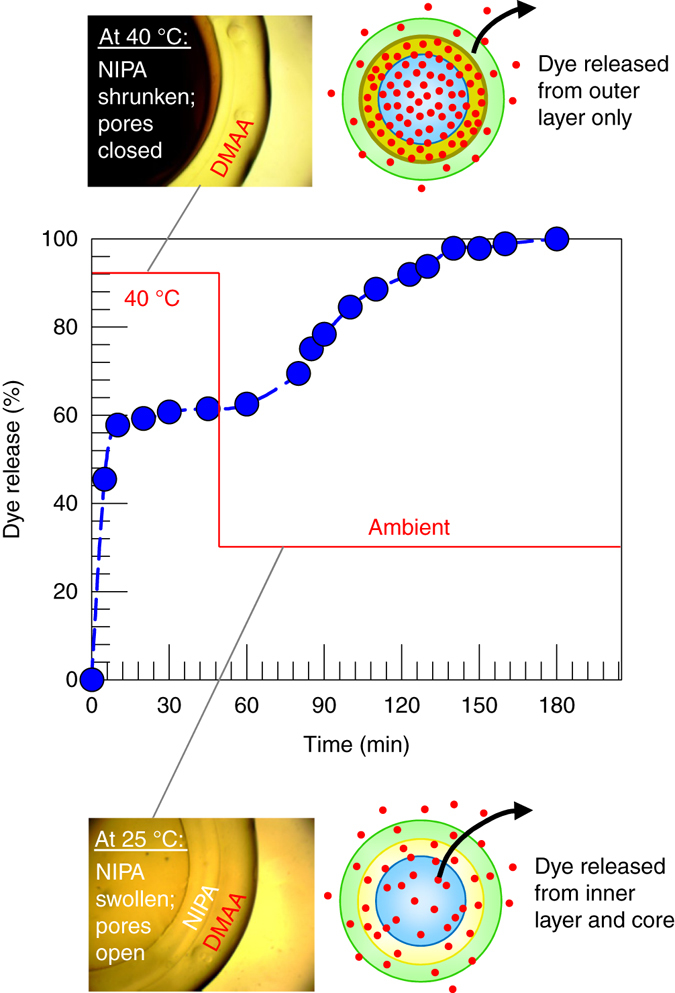
Temperature-responsive release of dye from a two-layer NIPA–DMAA capsule. Here, NIPA is the inner layer and DMAA is the outer layer. At 40 °C (above the LCST of NIPA), the pores in the inner NIPA layer are closed. In this case, the dye in the outer DMAA layer alone is released, as shown by the schematic. The corresponding micrograph shows a dark inner portion due to the turbidity of the NIPA layer, while the outer layer is transparent. At the 45 min mark, the temperature is lowered to ambient (25 °C). The pores in the NIPA layer open up, causing the inner dye to also release. This is shown by the schematic and in this case the entire capsule is transparent

Next, we consider an Alg–NIPA–DMAA capsule, i.e., with NIPA as the inner layer and DMAA as the outer layer. We again loaded the capsule with BY dye; note that some of the dye will be in the alginate core and inner NIPA layer, while some of it will be in the outer DMAA layer. The capsule was then transferred to a flask at 40 °C containing 100 ml of DI water. The release profile (Fig. 7) shows an initial rapid release of dye, followed by a saturation around the 45 min mark. This released dye corresponds to that in the outer DMAA layer. The dye in the core is prevented from diffusing out because the pores in the NIPA layer are closed at 40 °C. In the corresponding microscope image in Fig. 7, the core and NIPA layer appear black, but the DMAA layer is clear. When the heat is removed at the 45 min mark, the system cools to ambient temperature, and at this point the entire capsule appears clear. In turn, the pores in the NIPA layer are opened, allowing dye trapped within the core to be released. Thus, the release profile shows a second bump followed by a saturation at about the 180 min mark. The *y*-axis in Fig. 7 is normalized by the final dye concentration in the solution; thus, ~60% of the dye in the capsule is released at 40 °C, while the remaining 40% of the dye is released upon cooling. Overall, by changing the capsule architecture, i.e., order of layers, we have now been able to engineer a two-step release profile, with some of the solute being released at one value of the stimulus, and the rest of the solute being released at a different value of the stimulus.

## Discussion

We have demonstrated an inside-out technique for creating multilayered polymer capsules, with each layer being a crosslinked polymer gel. The technique is very simple to implement, and does not require complex multiphase precursors such as double emulsions, nor does it require strong interactions (electrostatic or hydrophobic) between adjacent layers. As a result, a wide variety of polymers can be used to form the layers. The technique uses a gelled core that is loaded with water-soluble initiator and then placed in a solution containing monomer, crosslinker and accelerant. The initiator diffuses out of the core into the surrounding solution, whereupon polymerization begins at the surface of the core. A polymer layer is formed by free-radical polymerization, and as time progresses, this layer grows outward. The process can be sequentially repeated with different monomers to generate additional layers. Layer thickness can be controlled based on the polymerization time or by varying the amount of initiator in the core.

The utility of this technique is shown by juxtaposing layers of a non-responsive polymer next to either a temperature or pH-responsive polymer. In these cases, the thickness of the stimuli-responsive layer can be altered substantially by varying the external stimulus while the non-responsive layer remains at the same thickness. In addition, the permeability of small molecules through the stimuli-responsive layers is also altered. For example, when NIPA is one of the layers, the release of a small-molecule dye from the capsule is very slow above the LCST of NIPA but much faster below the LCST. As a result, a two-layer capsule with an inner DMAA and outer NIPA layer shows a one-step release profile with varying temperature. Conversely, when NIPA is the inner and DMAA the outer layer, the capsule shows a two-step release when subjected to the same temperature profile. Thus, new modes of pulsatile release are made possible by these multilayer capsules.

The multilayer capsules described here can be extended in numerous ways. Unusual combinations of polymeric layers can be incorporated into a given capsule. Also, nanoparticles of different kinds can be incorporated into specific layers during synthesis. We have also briefly shown that these multilayer capsules can have remarkable mechanical properties; these also depend on the composition of each layer and on the number of layers. In addition, the optical properties of these capsules can also be tuned based on the content of each layer. We hope that the versatility of this technique will prove attractive to researchers. Many new kinds of multilayer capsules with expanded functionalities are envisioned in the future, with potential applications in fields, such as catalysis, biomimetics, and the delivery of drugs and therapeutics.

## Methods

### Materials

The monomers DMAA and N-isopropylacrylamide (NIPA), and the accelerant, N,N,N′,N′-tetramethylethylenediamine (TEMED) were from TCI America. All other chemicals were from Sigma-Aldrich, including the cross-linker N,N′-methylenebis(acrylamide) (BIS) and the monomer, SA. Three biopolymers were used: alginate, i.e., medium viscosity alginic acid, sodium salt from brown algae, chitosan (medium molecular weight), and xanthan gum (from *Xanthomonas campestris*). Other chemicals included calcium chloride dihydrate (CaCl_2_) salt, APS initiator, GA, glacial acetic acid, and BY dye. DI water was used in all the experiments.

### Synthesis of gel cores

To form the alginate gel cores, a 2 wt% alginate solution was first made in DI water. This was then added drop-wise using a transfer pipette or syringe into a solution of 0.5 M CaCl_2_ under mild stirring. After incubation for 30 min, Ca^2+^-cross-linked alginate gels were obtained. To form the chitosan gel cores, a 2 wt% chitosan solution was made in 0.2-M acetic acid. This was then added drop-wise (as above) to a solution of 2 wt% GA. After incubation for 24 h, chitosan gels crosslinked by GA were obtained. To form gel cores with diameters <1 mm, a pulsed-gas micro-capillary device was used^36, 37^. The biopolymer solution of interest was sent through a capillary of 80-µm inner diameter at a flow rate of 3 µl/min. Pulses of nitrogen gas (4 Hz frequency at 9 psi) were applied to the tip of the capillary, leading to the formation of microscale droplets, which were then cross-linked as above.

### Synthesis of multilayer capsules

Multilayer capsules were synthesized by the procedure described in the previous section (Fig. 1). First, the gel core prepared in the previous step was placed in an aqueous solution of 15 mg/ml APS initiator for 10 or more minutes. The gel was then removed from the solution and blotted with a Kimwipe to remove excess solution. The APS-soaked gel was then transferred into the desired monomer solution. Typically, the monomer (e.g., DMAA or NIPA) was at a concentration of 1 M. A cross-linker (typically BIS) was added at a concentration of 2.2 mol% with respect to the monomer. In addition, 15 mg/ml of the accelerant TEMED and 0.5–0.75 wt% of xanthan gum were added to the solution. The function of the TEMED was to accelerate the polymerization, thereby allowing it to be conducted at room temperature. The xanthan gum was used to increase the viscosity of the solution, which was important to keep the capsule suspended during polymerization. Thereafter, free-radical polymerization, initiated by persulfate ions from the APS, was carried out at room temperature. The time for polymerization was dictated by the time needed for the layer thickness to saturate, which was typically around 10–20 min (see Fig. 3). In many cases, polymerization was continued for a period of 24 h to allow ample time to reach a steady state. Once the first layer was formed, the polymer capsule was washed with water and stored in DI water. To form the next layer, the above procedure was repeated, as shown in Fig. 1. In the case of the pH-responsive multilayer capsules shown in Fig. 5, a mixture of DMAA (nonionic) and SA (anionic) in a molar ratio of 9:1 DMAA:SA was used for the pH-responsive layer, with the total monomer concentration being 1 M as above.

### Optical microscopy

Bright field images of capsules were captured with a Zeiss Axiovert 135 TV microscope. Images were taken using either a ×2.5 or a ×10 objective. In some cases, the microscopy was performed with slight under-focus, which helped to clearly define the outlines of the layers and/or the overall capsule.

### Scanning electron microscopy (SEM)

A two-layer capsule with an inner layer of NIPA and an outer layer of DMAA was frozen rapidly in a −80 °C freezer, and subsequently lyophilized. Next, the capsule was fractured with a razor and affixed to a viewing platform. The capsule was then sputter coated with gold. A Hitachi SU-70 Schottky field emission SEM was used to obtain images of the sample.

### Compression tests

An AR 2000 stress-controlled rheometer (TA Instruments) was used to conduct the compression tests at 25 °C. From the rheometer software, the squeeze-test mode was chosen, and the tests were done using steel parallel plates (40 or 20 mm diameter)^40, 41^. The spherical sample of interest (gel or capsule) was placed at the center of the plates. Compression was done at a rate of 10% strain per minute, which was determined based on initial sample diameter. The plates were coated with a thin layer of mineral oil to avoid excessive adhesion to the samples during compression. The normal-stress transducer was used to collect the normal force during compression, and this was converted to stress based on the initial surface area of the capsule.

### Controlled release experiments

For the dye release studies (Figs. 6 and 7), capsules were loaded with BY dye by soaking in a 500 μM dye solution for 24 h. Capsules were then added to 100-ml Erlenmeyer flasks filled with DI water, and the flasks were placed in a temperature-controlled water bath (Julabo). To monitor the dye concentration, a 1.5-ml sample was taken every 10 min from the supernatant surrounding the capsule, and this was analyzed using a Cary 50 UV-Vis spectrophotometer. After analysis, the sample was returned to the flask containing the capsule.

### Data availability

The data that support the findings of this study are available from the corresponding author upon request.

## Electronic supplementary material

Supplementary Information

Supplementary Movie 1

Supplementary Movie 2
