# Supplementary material for: Onion-like multilayered polymer capsules synthesized by a bioinspired inside-out technique
Source: Nat Commun. 2017 Aug 4;8:193. doi: 10.1038/s41467-017-00077-7 (PMC5544678; doi:10.1038/s41467-017-00077-7)
Supplement: Supplementary file 1 — Supplementary Information [file 41467_2017_77_MOESM1_ESM.pdf]

File Name: Supplementary Information
Description: Supplementary Figures

File Name: Supplementary Movie 1
Description: Compression Test on Alg Gel Core

File Name: Supplementary Movie 2
Description: Compression Test on Alg-DMAA Capsule

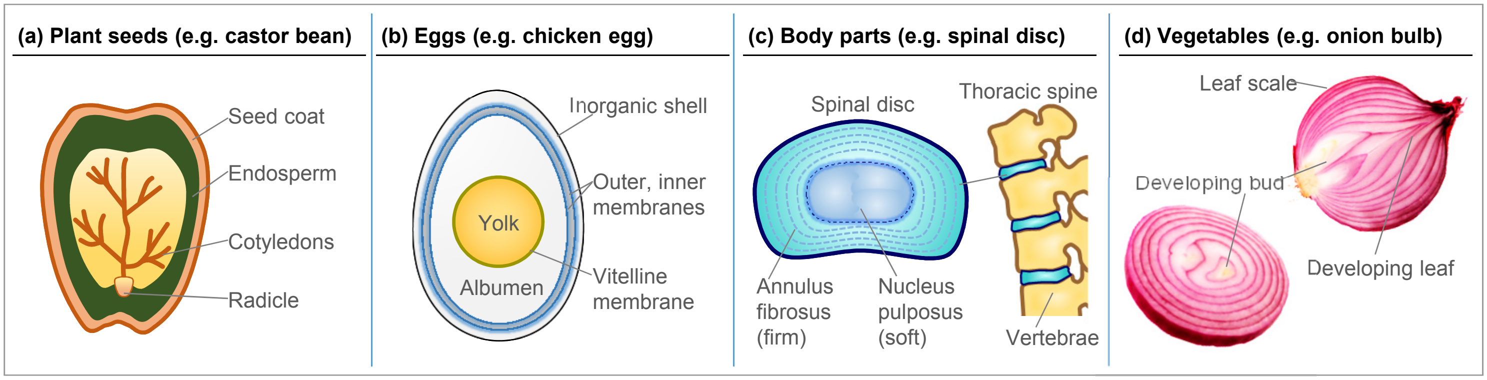

Supplementary Figure 1. Examples of natural materials that have multiple, roughly concentric, layers. (a) Plant seeds. The cross-section of a castor bean seed is shown. (b) Eggs and embryos. The structure of a chicken egg is illustrated. (c) Tissues and body parts. The top-view of a spinal disc located between the vertebrae in the spine is shown. (d) Vegetables and fruits. The structure of an onion is shown in two views. In the above schematics, different layers in each material are labeled. Note that the composition of each layer is tied to its distinct function in the overall material.

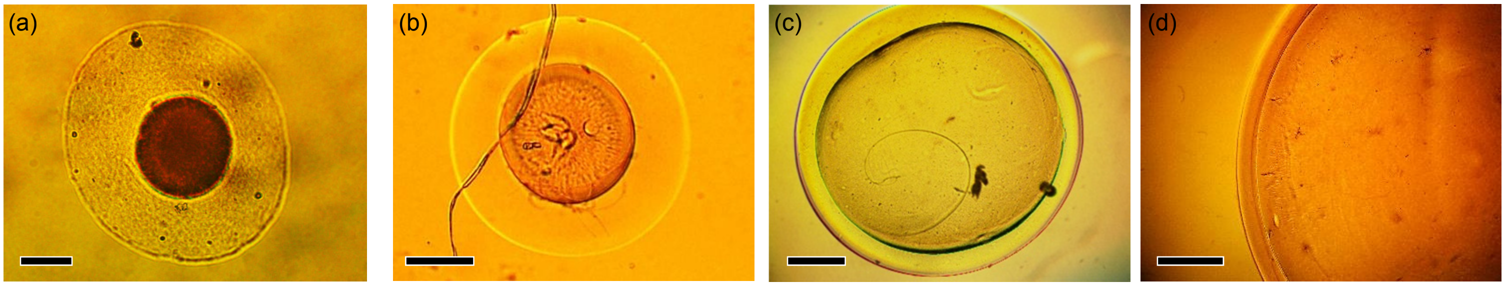

Supplementary Figure 2. Single-layer capsules over a range of length scales. Optical micrographs are shown of capsules with a crosslinked polymeric shell surrounding a biopolymer gel core. In (a), the core (chitosan/GA) has a diameter of 185 µm, and the shell (SA) has a similar thickness (scale bar is 100 µm). In (b), the core (chitosan/GA) has a diameter of 400 µm and the shell (DMAA) has a thickness of ~ 150 µm (scale bar is 200 µm). In (c), the core (alginate) has a diameter of 2.8 mm and the shell (DMAA) is 250 µm thick (scale bar is 700 µm). In (d), the core (alginate) has a diameter of 6.0 mm and the shell (DMAA) is 230 µm thick (scale bar is 800 µm).

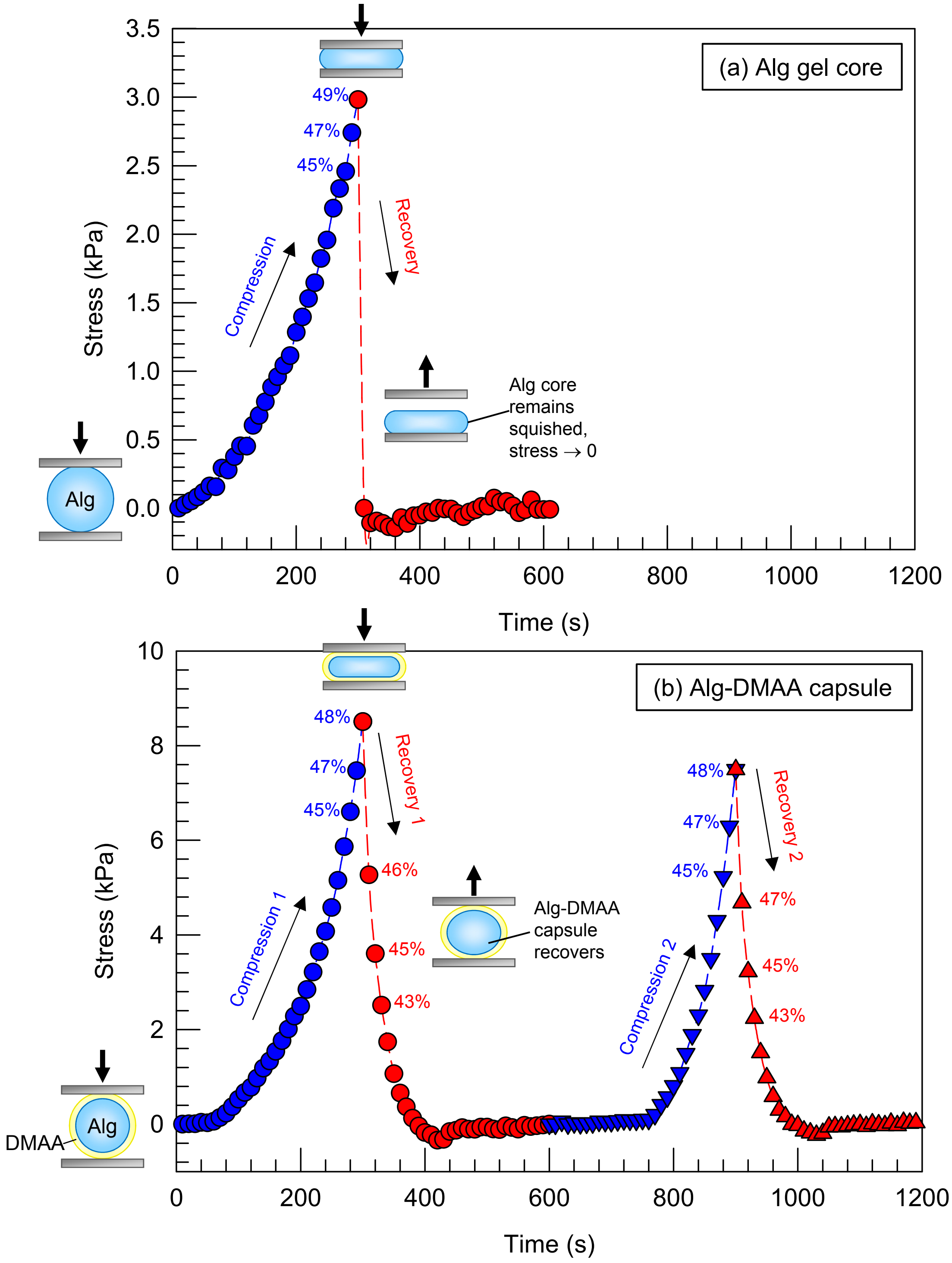

Supplementary Figure 3. Compression tests on an alginate (Alg) gel core (a) and an Alg-DMAA capsule (b). The sample is placed between parallel plates at time zero. During the compression cycle the top plate is brought down, while during the recovery cycle the top plate is lifted up (both at 10% strain per minute). The measured compressive stress is plotted against time in the two plots, with the compressive strain indicated for selected points. (a) When the Alg gel core is compressed up to ~ 50% strain, it is irreversibly squished into a disc shape (plastic behavior). Thus, the sample does not recover when the plate is lifted up. (b) When the Alg-DMAA capsule is compressed up to ~ 50% strain, it responds elastically, and recovers to its initial size when the plate is lifted up during the recovery cycle. A second compression-recovery cycle is then applied on the capsule, and the data for this cycle closely track those from the first cycle.
